# Clonal divergence and genomic meltdown in prostatic pleomorphic giant cell adenocarcinoma

**DOI:** 10.3389/fimmu.2025.1609340

**Published:** 2025-08-12

**Authors:** Xiaoshi Ma, Kun Chen, Jing Zhang, Liming Liu, Jiping Luo, Kaipeng Huang, Hongying Zhang, Danni Liu, Jizhou Gou, Changyin Feng, Xia Zhao, Wanying Li, Lipeng Chen, Li Yin, Xianlin Meng, Zhiqiang Cheng

**Affiliations:** ^1^ Department of Pathology, Shenzhen Third People’s Hospital (The Second Affiliated Hospital of Southern University of Science and Technology), Shenzhen, Guangdong, China; ^2^ Department of Urology, Shenzhen People’s Hospital (The First Affiliated Hospital of Southern University of Science and Technology), Shenzhen, Guangdong, China; ^3^ Cancer Hospital Affiliated to Nanjing Medical University and Jiangsu Cancer Hospital and Jiangsu Institute of Cancer Prevention and Control, Nanjing, Jiangsu, China; ^4^ Department of Radiation Oncology, The Fourth School of Clinical Medicine of Nanjing Medical University, Nanjing, Jiangsu, China; ^5^ Department of Bioinformatics, HaploX Biotechnology, Shenzhen, Guangdong, China; ^6^ Department of Pathology, Shenzhen People's Hospital (The First Affiliated Hospital of Southern University of Science and Technology), Shenzhen, Guangdong, China; ^7^ Department of Experimental Research, State Key Laboratory of Oncology in South China, Sun Yat-sen University Cancer Center, Guangzhou, Guangdong, China; ^8^ Collaborative Innovation Center of Tumor Individualized Medicine, Nanjing Medical University, Nanjing, Jiangsu, China; ^9^ Department of Laboratory, Tianjin Fifth Central Hospital (Peking University BinHai Hospital), Tianjin, China

**Keywords:** prostate cancer genomics, pleomorphic giant cell adenocarcinoma, divergent tumor evolution, cell cycle, apoptosis, therapeutic resistance biomarkers

## Abstract

**Background:**

Pleomorphic giant cell adenocarcinoma (PGCA) of the prostate is a rare, aggressive variant characterized by multinucleated giant cells, sarcomatoid features, and resistance to conventional therapies. Despite its recognition in the WHO 2016 guidelines, the molecular drivers and clinicopathological correlates of PGCA remain poorly characterized. This study presents the first integrative clinicogenomic profiling of PGCA, revealing a novel prognostic gene signature with direct implications for diagnosis and treatment.

**Methods:**

We conducted comprehensive clinicopathological and genomic analyses of a treatment-refractory PGCA case using histology, immunohistochemistry (IHC), whole-exome sequencing (WES), clonal evolution modeling, and multicohort validation. IHC assessed key prostate cancer markers (AR, AMACR, KLK3, PTEN, NKX3-1, VIM), while WES compared somatic alterations in PGCA, adjacent adenocarcinoma, and stromal tissue. Public datasets (prostate_dkfz_2018, prad_tcga, prad_mcspc_mskcc_2020) were used for external validation.

**Results:**

PGCA displayed profound pleomorphism, necrosis, and complete loss of luminal markers (AR/AMACR/KLK3), along with strong vimentin (VIM) expression, consistent with epithelial–mesenchymal transition. WES revealed PGCA-specific mutations enriched in cell-cycle and inflammatory response pathways, distinct from metabolic alterations in the adjacent adenocarcinoma. Clonal evolution analysis showed divergent progression from a shared ancestral clone. Importantly, mutations in ADAMTS7, CDH1, DRD5, MGAT5, and TP53 emerged as a robust five-gene signature predictive of biochemical recurrence, metastasis, and poor survival, validated across multiple independent cohorts.

**Conclusion:**

Our study provides the first molecular roadmap of prostatic PGCA to date, establishing a novel five-gene prognostic signature and revealing fundamental insights into its pathogenesis through divergent evolution from conventional adenocarcinoma. These insights offer new opportunities for precise diagnosis, prognostic stratification, and targeted therapeutic strategies for this lethal prostate cancer variant.

## Introduction

Pleomorphic giant cell adenocarcinoma (PGCA) is a rare and highly aggressive malignancy that has been identified in various organs, including the lung, pancreas, thyroid, bladder, and prostate (1–5). In the context of prostate, PGCA is histologically characterized by the presence of bizarre multinucleated giant cells, marked nuclear atypia, and sarcomatoid differentiation, setting it apart from the conventional prostatic adenocarcinoma (6). With the increasing number of similar cases reported in prostate, PGCA was classified by the World Health Organization (WHO) 2016 as a variant of prostatic adenocarcinoma (7). Clinically, prostatic PGCA manifests with non-specific symptoms such as urinary obstruction, hematuria, and pelvic pain, yet distinguishes itself through rapid progression, early metastasis to bones and visceral organs, and a median survival of less than 12 months post-diagnosis (8). In comparison, patients with conventional prostatic adenocarcinoma harboring high-grade Gleason scores (9, 10) exhibit a mortality rate of less than 10% within 4 years post-diagnosis following standard therapeutic interventions (9, 10).

Therapeutic management of prostatic PGCA remains a significant challenge due to its intrinsic resistance to conventional treatments for prostate cancer (PCa), including androgen deprivation therapy (ADT), chemotherapy, and radiation (11). Moreover, previous treatments may even be responsible for the clonal evolution of PGCA, particularly ADT (6). Compounded by the inefficacy of immunotherapy in most advanced PCa cases (12–14), no currently therapeutic options can bring progression-free survival (PFS) and overall survival (OS) benefits. As an exceptionally rare entity, only approximately 50 cases have been reported in the medical literatures, accounting for <0.1% of all prostatic malignancies (15). Given this rarity, prostatic PGCA often presents significant treatment challenges, exacerbated by its aggressive nature and poor prognosis, underscoring the need for more comprehensive data on this rare pathologic entity.

Histopathological analyses reveal a high mitotic index, extensive necrosis, and heterogeneous cellular architecture, features that correlate with its aggressive phenotype (15, 16). Limited available molecular data have identified a variety of gene alterations such as PTEN loss; TP53, BRAC2, and PIK3CA mutation; and TMPRSS2–ERG fusion as the genomic signature of PGCA (6, 11, 17, 18). However, a comprehensive genomic landscape of PGCA remains sparse, with the limited gene alteration information obtained mostly from gene panel sequencing. Notably, another critical barrier for better understanding the mutational landscape of PGCA besides the scarcity is the profound tumor heterogeneity, evidenced by the coexisting adenocarcinoma, more differentiated, yet high-grade (8, 19). Such heterogeneity not only fuels therapeutic resistance but also complicates the molecular mechanism underneath (20).

In the present study, we examined the histology characteristics of a prostatic PGCA patient with bone metastasis through H&E staining and immunohistochemical profiling. To delineate the mutational landscape of PGCA precisely, laser-capture microdissection was employed to isolate specific regions, including PGCA, conventional adenocarcinoma, and adjacent stromal compartments for whole-exome sequencing (WES). Functional enrichment analysis and clonal evolution modeling based on somatic mutations were performed to interrogate dysregulated biological pathways and the mechanistic origins of PGCA. Gene mutations significantly correlated with dismal prognosis in PGCA were identified and further verified with the transcriptional profiles of PCa patients from public datasets. Our findings revealed the distinct molecular signatures of PGCA and coexisting adenocarcinoma, uncovering potential driver mutations that may underlie PGCA pathogenesis and confer resistance to current therapies.

## Materials and methods

### Patient characteristics and tissue samples

One 74-year-old Chinese man with elevated PSA for a month was admitted to Shenzhen People’s Hospital in March 2019. Digital rectal examination (DRE) showed an obvious intrarectal lump, and then the patient underwent needle biopsy. A total of 12 biopsy samples were collected from different spots of prostate. After a series of treatments, the samples were embedded in paraffin and further sectioned for H&E staining. Two qualified pathologists diagnosed this prostatic disorder as PGCA according to the morphology alterations. Furthermore, bone scan showed potential metastasis in multiple spots. The patient received hormone therapy for 3 months with a second elevation of PSA and then took chemotherapy additionally. Eventually the patient underwent TURP and received the combination of hormone therapy and chemotherapy. The resected samples were consistently treated as before and performed H&E staining and gene mutation analysis.

### Immunohistochemical staining

Needle biopsy and transurethral resection samples were sectioned for immunohistochemical staining to explore the expression characteristics of PCa pathology markers. The sections were immersed in 3% H_2_O_2_ for 10 min at room temperature to block endogenous peroxidase and incubated in citrate buffer at 95°C for 40 min for antigen retrieval. After being blocked with 3% bovine serum albumin for 1 h at room temperature, they were incubated by primary antibodies anti-AR (diluted at 1:500, Abcam, cat. no. ab133273), AMACR (diluted at 1:100, Abcam, cat. no. ab194396), KLK3 (diluted at 1:1,000, Abcam, cat. no. ab76113), NKX3-1 (diluted at 1:500, Abcam, cat. no. ab196020), PTEN (diluted at 1:2,000, Abcam, cat. no. ab267787), and VIM (diluted at 1:500, Abcam, cat. no. ab92547) at 4°C overnight. HRP-linked secondary antibody (Abcam, cat. no. ab7090) incubation was performed subsequently followed by 3,3′-diaminobenzidine (DAB) staining. The sections were counterstained with hematoxylin to detect nucleus.

### Whole-exome sequencing

To obtain relatively precise genomic information of PGCA, we collected different tissue areas mainly consisting of pleomorphic giant cells, adenocarcinoma cells, and stromal cells by Applied Biosystems ArcturusXT™ LCM. The transurethral resected samples were sectioned and put on the special slide used for LCM. After H&E staining, we performed LCM to collect PGCA, PCa, and stroma samples from various spots. Genomic DNA of these samples were isolated by QIAamp DNA FFPE Tissue Kit (QIAGEN, cat. no. 56404) according to the manufacturer’s instructions and sent to Genergy Biotechnology Inc., Shanghai, China, for whole-exome sequencing (WES).

### Data processing and mapping

Raw sequencing data were processed by fastp (v0.12.6) as the following criteria: 1) removing adaptors; 2) removing reads with more than five uncertain bases (N); 3) removing reads with more than 40% low quality bases (phred quality score ≤20); 4) sliding window trimming (four bases). Clean reads were mapped to the reference GRCh37/hg19 genome using the BWA mem function. BAM files were sorted by the Sentieon tools (v201808), and the duplicate reads were removed using Sambamba (v0.6.6).

### Gene mutation annotation

Aligned reads were further processed with GATK (v.4.1.1.0) for base quality recalibration. Somatic mutations were determined via MuTect2 (v4.1.1.0) and annotated by ANNOVAR (v2018-04-16). Those outside the target region, supported by <3 reads or covered by <10 reads, were disregarded. Low-confidence somatic mutations considered likely germline in ESP6500, 1000 Genomes, or ExAC_EAS (global minor allele frequency >0.1%) were also dismissed, excluding those with presence in the COSMIC database. In addition, synonymous mutations were filtered out for further analyses. R package ComplexHeatmap (v1.20.0) was used to visualize the mutational landscape.

### Clonal evolution analysis

The evolutionary relationships and temporal order of each area collected from PGCA samples during disease progression were evaluated by cellular frequency (CCF) of gene mutations using PyClone-VI (v0.1.0). Clusters containing at least five mutations were used to infer a consensus clonal evolution model using ClonEvol.

### Gene functional enrichment analysis

Mutated genes specific in pleomorphic giant cells and adenocarcinoma cells compared with stroma were identified. Functional enrichment analysis was performed using Fisher’s exact test as implemented in the clusterProfiler package (v3.8.1), with a Bonferroni correction and an adjusted p-value of 0.05.

### Clinical correlation analysis

Gene mutations and clinical information of the PCa patients from public datasets were downloaded from cBioPortal. The cBioPortal datasets used in this analysis include “Prostate Cancer (DKFZ, Cancer Discov 2018),” “Prostate Adenocarcinoma (TCGA, PanCancer Atlas),” “Prostate Cancer, Metastatic Castration-Sensitive (MSKCC, Cancer Cell 2020),” and “Prostate Cancer, PI3K Pathway Alterations” (MSKCC, Nat Med 2021). To estimate independent prognostic factors from PGCA mutations, we constructed a Cox proportional hazards model with genomic and prognosis information from dataset prostate_dkfz_2018. Other public datasets were divided into mutant and wild type by candidate gene mutations for PFS and OS analysis to verify their clinical relevance to adverse prognosis. Furthermore, we used a chi-square (χ2) test to examine the correlation of candidate gene mutations with pathology grade (Gleason score) and metastatic potentials. DEGs between samples from mutation carriers and wild-type PCa patients provided by public datasets were identified with Linear Models for Microarray Data (limma) as the FDR-adjusted p-value <0.05 and fold change >2. Functional enrichment of these DEGs was analyzed as the same method above.

## Results

### Distinct morphological and immunohistochemical characteristics of PGCA

**Figure 1:**
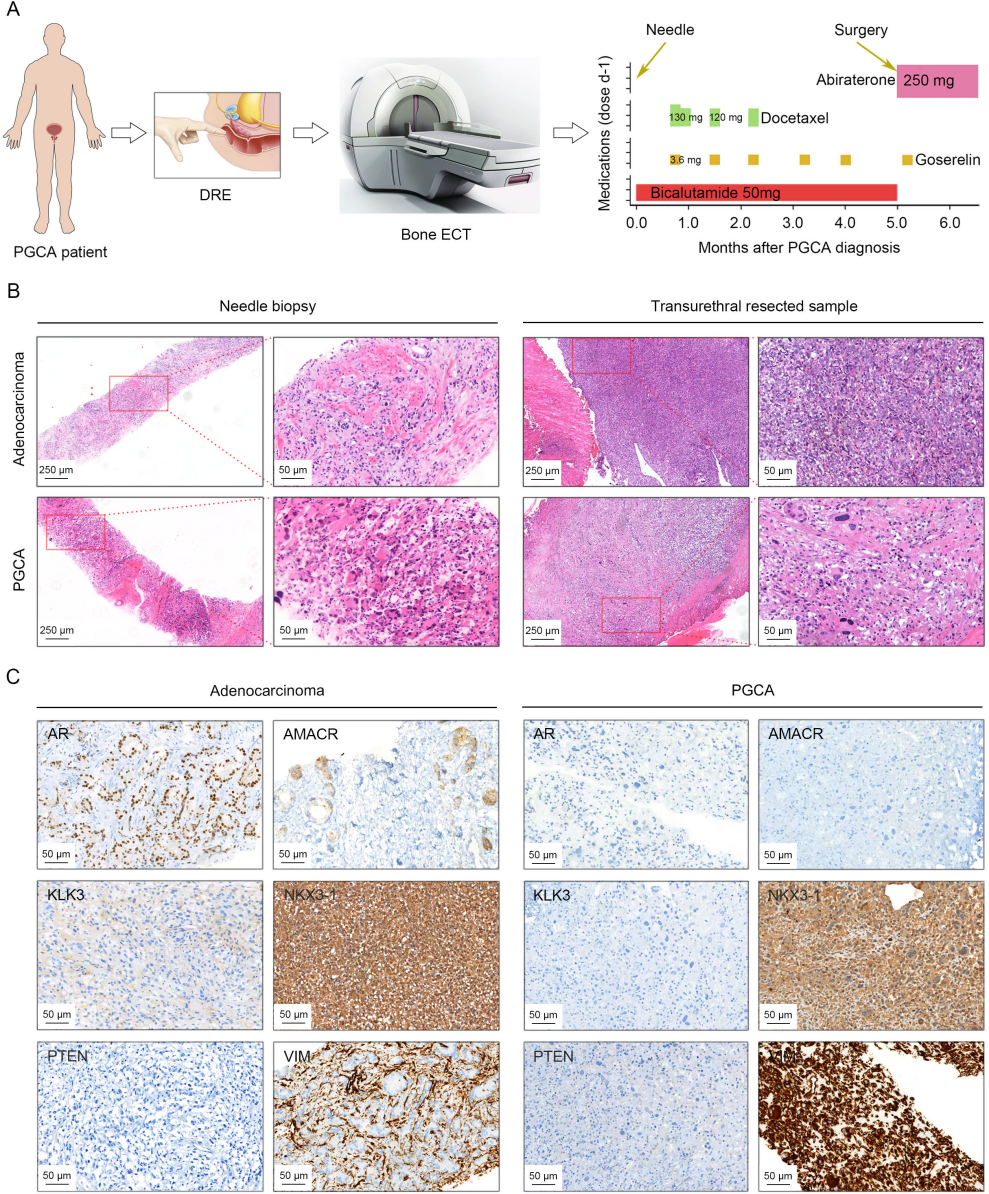
Histopathology characterization of PGCA and coexisting adenocarcinoma by H&E staining and immunohistochemical evaluation. **(A)** Schematic drawing illustrating diagnostic procedures and therapeutic interventions for the PGCA case, including pharmacological management and surgical resection. **(B)** Histopathology examination of biopsy and surgical samples from PGCA and adenocarcinoma components. **(C)** Immunohistochemical staining demonstrating the expression characteristics of AR, AMACR, KLK3, NKX3-1, PTEN, and VIM in PGCA and adenocarcinoma. Positive signals with different antibodies were stained in brown. The cell nucleus was stained with hematoxylin and presented blue.

A patient with elevated serum prostate-specific antigen (PSA) for a whole month underwent DRE and bone emission computed tomography (ECT), suggesting a possible prostatic neoplasm with multiple bone metastases (**Figure 1A**). The morphology observations from needle biopsies confirmed the presence of both conventional adenocarcinoma and PGCA components (**Figure 1A**). The patient was administered with systemic therapies including chemotherapy (docetaxel) and endocrinotherapy (goserelin and bicalutamide) for 5 months, followed by transurethral resection of the prostate (TURP) due to the massive tumor burden and the replacement of bicalutamide with abiraterone (**Figure 1A**). Despite aggressive treatment, the patient exhibited disease progression and died within 6 months post-diagnosis, underscoring the aggressive nature of PGCA. Needle biopsies and transurethral resected samples were both performed H&E staining for histological analysis, demonstrating distinct morphological features of PGCA. The adenocarcinoma component showed typical glandular structures with relatively uniform nuclei and moderate cytoplasm (**Figure 1B**). In contrast, PGCA exhibited significant cellular pleomorphism, abundant multinucleated giant cells and extensive necrosis, showing a more aggressive histological phenotype (**Figure 1B**). To further explore the cellular and molecular basis of PGCA pathogenesis, we performed immunohistochemical staining with key PCa markers and found that AR and AMACR were highly expressed in the adenocarcinoma component, consistent with luminal differentiation, yet no expression was found in PGCA, indicative of lineage plasticity (defined as the ability of cancer cells to switch lineage identity in response to therapeutic pressure or microenvironmental cues, is increasingly recognized as a mechanism of treatment resistance and tumor progression in prostate cancer) and dedifferentiation (**Figure 1C**). As one of the downstream genes of AR, KLK3 was also negatively stained in PGCA, suggesting that the elevation of PSA was derived from the coexisting adenocarcinoma (**Figure 1C**). Loss of PTEN and amplification of NKX3-1, the representative hallmarks of PCa progression, were found both in PGCA and adenocarcinoma components (**Figure 1C**). Additionally, VIM was highly expressed in the densely fibrotic stroma of adenocarcinoma region, not in the tumor cells (**Figure 1C**). In contrast, the positive signal of VIM staining was significantly enriched in the pleomorphic giant cells of the PGCA region, suggesting a transition to a more primitive, treatment-resistant phenotype (**Figure 1C**). These findings indicated that the PGCA component was more aggressive and likely to be the fundamental element for therapeutic resistance and adverse prognosis.

### Mutational landscape of PGCA and its aberrant enrichment cell cycle and apoptosis

**Figure 2:**
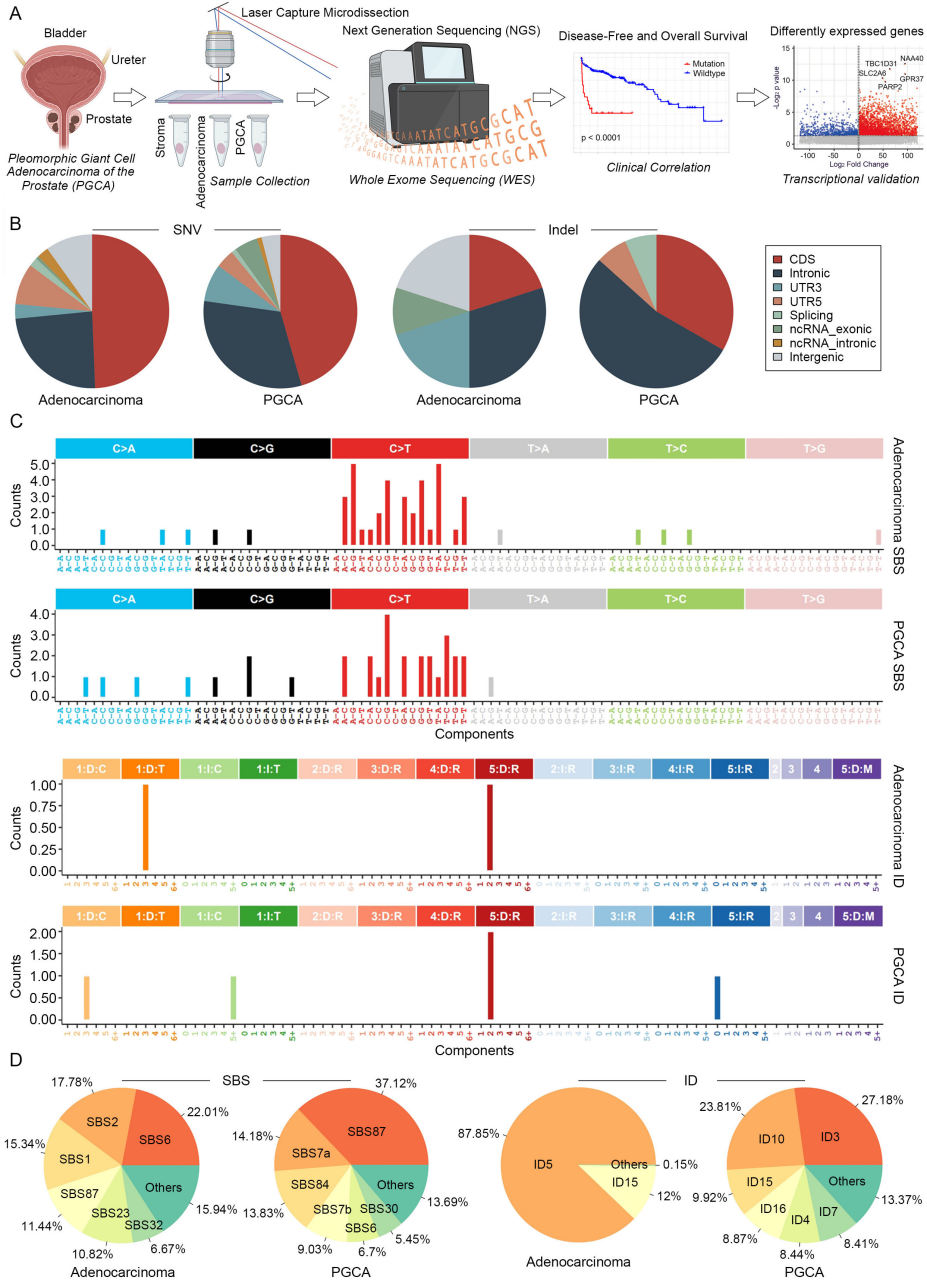
Mutational signatures of PGCA and adenocarcinoma revealed by WES. **(A)** Experimental design of genomic analysis and transcriptional validation. LCM-isolated PGCA, adenocarcinoma, and stroma components from TRUP to perform WES. Prognostic gene mutations identified by PFS and OS analyses and further validated by transcriptional profiles from public datasets. **(B)** Proportional distribution of SNVs and indels across genomic regions. **(C)** Bar plot showing the mutation signatures of PGCA and adenocarcinoma. **(D)** Pie charts illustrating compositional differences in mutation signatures.

To precisely elucidate the mutational profiles of prostatic PGCA, we performed WES on microdissected samples, including PGCA, coexisting adenocarcinoma and stroma (**Figure 2A**). Genes mutated specifically in the PGCA component and significantly associated with dismal prognosis were identified and further validated with transcriptional profiles (**Figure 2A**). Mutations including single-nucleotide variants (SNVs) and insertions/deletions (indels) across genomic regions showed that PGCA exhibited a higher proportion of mutations in gene expression-related regions and fewer mutations in intergenic regions, demonstrating a significantly elevated mutational burden in PGCA (**Figure 2B**). The mutational signature analysis showed a notable prevalence of C > T base substitution in both PGCA and adenocarcinoma components, suggesting potential DNA repair deficiencies or treatment resistance mechanisms (**Figure 2C**). This C>T transition pattern is a hallmark of spontaneous 5-methylcytosine deamination, often linked to aging, oxidative stress, or treatment-induced DNA damage (21). Its prominence suggests potential defects in DNA repair mechanisms or therapeutic pressure-driven mutagenesis that may contribute to the aggressive behavior of PGCA. With the background the mutations in stroma, we found a striking predominance of Signature87 (SBS87, thiopurine chemotherapy) in PGCA, suggesting a potential association with resistance to DNA-damaging therapies (**Figure 2D**). Furthermore, indel signatures ID5, correlated with the age of cancer diagnosis, was significantly enriched in adenocarcinoma and also reported may accumulate in normal cells (**Figure 2D**). In contrast, the indel signatures involved in DNA mismatch repair deficiency, including ID3, ID4, ID7, and ID16, were exclusive to PGCA, further underscoring the genomic instability (**Figure 2D**).

**Figure 3:**
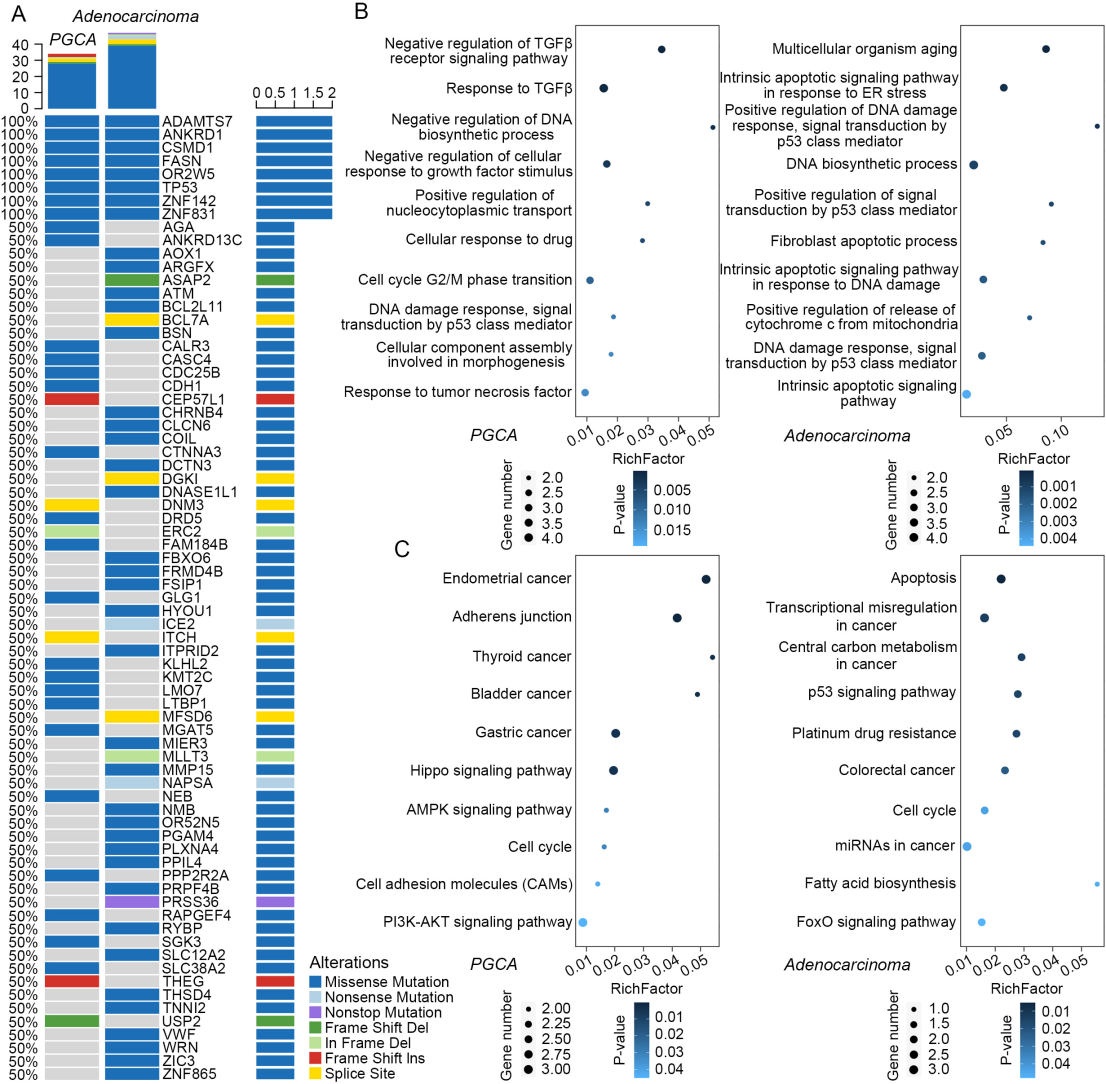
Mutational profiles and functional enrichment analysis. **(A)** Mutational landscape of PGCA and adenocarcinoma compared with stromal tissue. Rows represented mutated genes and sorted according to the frequency of mutations. Columns represent different legions of PGCA and adenocarcinoma. Different colors represent different types of mutations. **(B)** GO terms and **(C)** KEGG pathways enriched in PGCA and adenocarcinoma mutations.

Particularly, somatic mutations in ANKRD1, FASN, and TP53 were identified in both PGCA and adenocarcinoma components, with these alterations demonstrating significant associations with adverse prognosis in PCa (**Figure 3A**). The mutated genes in PGCA were predominantly enriched in cell cycle dysregulation, such as negative regulation of DNA biosynthetic process and cell-cycle G2/M phase transition, explaining the presence of bizarre multinucleated giant cells in PGCA by mitotic failure (**Figures 3B, C**). Furthermore, they were also involved in inflammatory factor response with potential correlation with apoptosis and immunosuppression, including TP53-related DNA damage response, response to inflammatory factors like TGFβ and TNF, and negative regulation of the TGFβ receptor signaling pathway, collectively contributing to enhanced DNA damage tolerance, immunosuppressive microenvironment, and therapeutic resistance (**Figures 3B, C**). In contrast, adenocarcinoma exhibited mutational enrichment in TP53-regulated apoptosis, such as positive regulation of DNA damage response, signal transduction by the p53 (tumor suppressor protein coded by TP53 gene) class mediator, and positive regulation of the signal transduction by p53 class mediator, suggesting a survival advantage under therapeutic pressure (**Figures 3B, C**). Furthermore, adenocarcinoma showed marked aberrations in central carbon metabolism and fatty acid biosynthesis, both of which are well-established contributors to malignant progression. Central carbon metabolism, particularly through enhanced glycolysis (Warburg effect), provides rapidly proliferating tumor cells with energy and biosynthetic precursors. Aberrant fatty acid biosynthesis, often driven by upregulation or mutation of FASN, promotes membrane biosynthesis, oncogenic signaling, and lipid-mediated protection from oxidative stress, supporting tumor growth and resistance to apoptosis (**Figures 3B, C**) (22–24). Taken together, the mutational landscape of PGCA was defined by defects in cell-cycle regulation and immune response pathways, whereas the coexisting adenocarcinoma displayed abnormalities in metabolic signaling and p53-mediated apoptosis. These molecular distinctions underline the divergent biological behaviors and clinical outcomes of PGCA and conventional prostate adenocarcinoma.

### Clonal evolution of heterogeneous PGCA and coexisting adenocarcinoma

**Figure 4:**
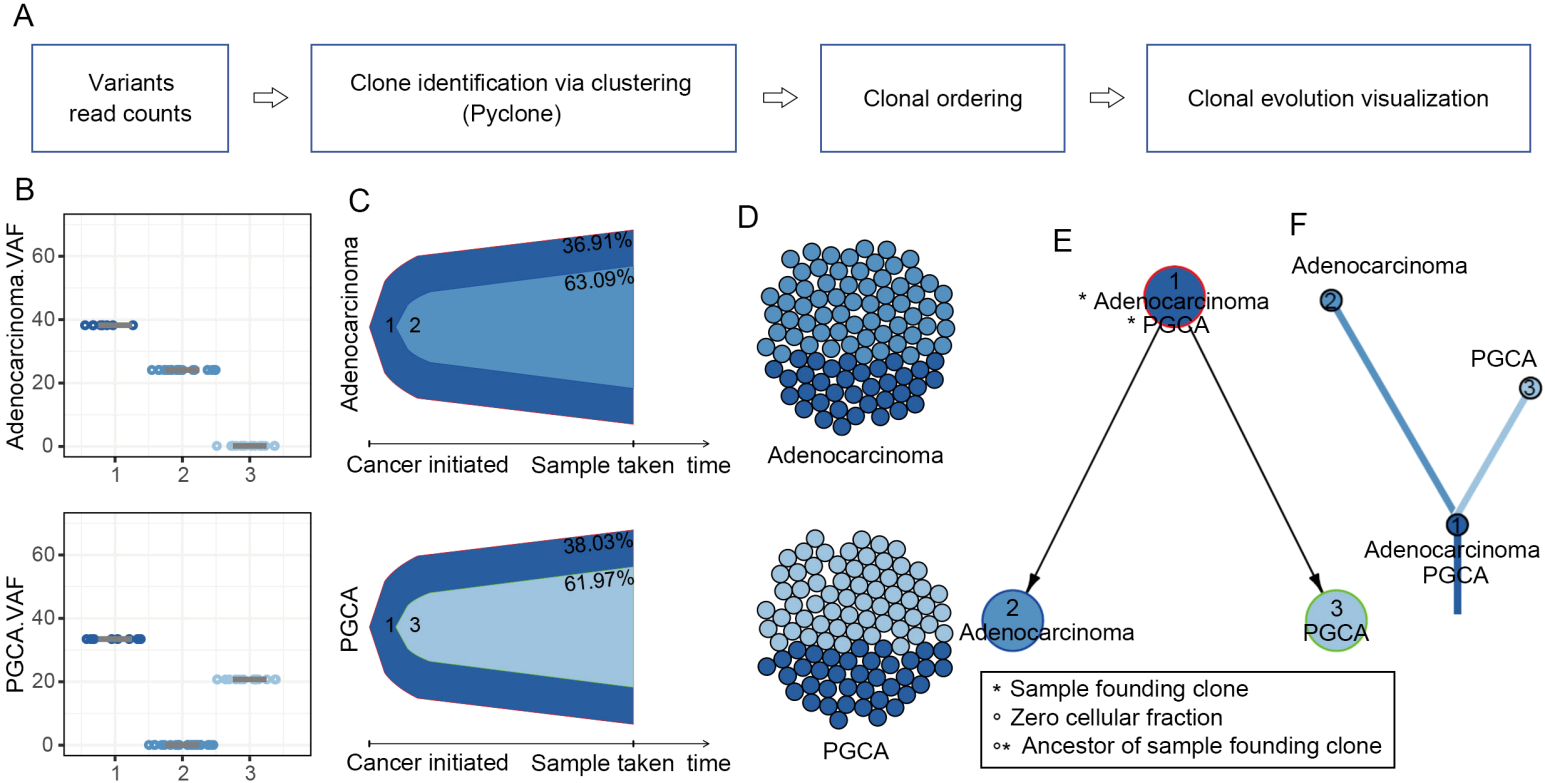
Clonal evolution patterns in PGCA and adenocarcinoma components. **(A)** Clonal evolution inference pipeline. **(B)** Boxplot comparing variant allele frequency (VAF) of each cluster. **(C)** Bell plot showing that the mutation signatures changed over the pseudo-tumor progression. **(D)** Spheres of cells presenting clonal subpopulations. **(E, F)** Node-based and branch-based trees depicting the clonal relationships of PGCA and adenocarcinoma.

Given that the adenocarcinoma components survived the aggressive treatments like PGCA, and their mutational enrichment in TP53-regulated apoptosis, we suspected if these malignant cells could be the precursor cells or daughter cells of PGCA. Therefore, we conducted a comprehensive analysis of clonal evolution in PGCA and the coexisting adenocarcinoma (**Figure 4A**). The clonalities of mutations represented by the variant allele frequency (VAF) distributions were divided into a total of three clusters (**Figure 4B**). Both of the PGCA and adenocarcinoma components contained cluster 1, making it the founding clone (**Figures 4C, D**). The adenocarcinoma contained cluster 2 uniquely, and the cluster 3 were specifically found in PGCA, indicating distinct clonal hierarchies between PGCA and coexisting adenocarcinoma (**Figures 4C, D**). The temporal evolution trajectory of clonal dynamics revealed a linear evolution pattern of both components that the ancestor clone 1 developed into two branches across time, with the end of clone 2 as the adenocarcinoma and clone 3 as the PGCA (**Figures 4E, F**). These findings revealed that PGCA and adenocarcinoma had the same origin and developed into two different forms of tumor cells during the malignancy progression.

### Identification of PGCA mutations associated with adverse prognosis

**Figure 5:**
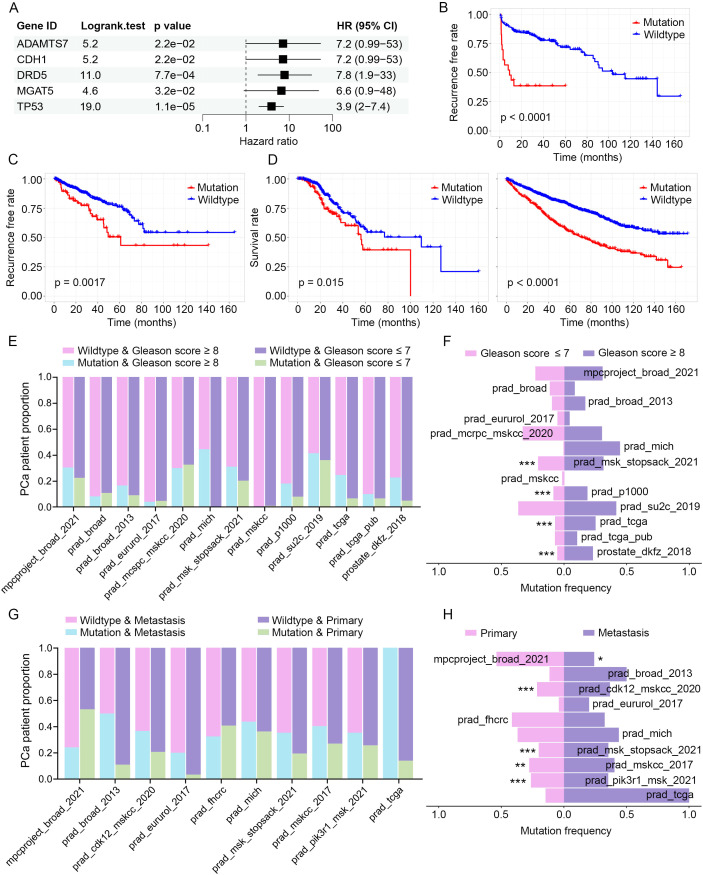
Clinical correlation of PGCA mutations in PCa cohorts. **(A)** Cox regression analysis identifying mutations associated with biochemical recurrence (prostate_dkfz_2018 dataset). **(B, C)** Kaplan–Meier curves predicting the recurrence-free rates of PCa patients with signature mutations (prostate_dkfz_2018 and prad_tcga dataset). **(D)** OS analysis with PCa patients based on the signature mutations (prad_mcspc_mskcc_2020 and prad_pik3r1_msk_2021 dataset). **(E)** Pathology grades (Gleason score ≥8 and ≤7) distribution stratified by mutation status. **(F)** Mutation prevalence across pathology grades (Gleason score ≥8 and ≤7). **(G)** Metastasis incidence by mutation status. **(H)** Mutation frequency comparison between primary and metastatic tumors. * p < 0.05, ** p < 0.01, *** p < 0.001.

To explore the potential clinical relevance of mutations identified in PGCA, we first analyzed the somatic mutations detected in PGCA via cBioWES and focused on those with likely roles in tumor progression. Given the limited number of PGCA cases and associated clinical follow-up, we employed the prostate_dkfz_2018 dataset—comprising a larger prostate cancer cohort with comprehensive genomic and prognostic data—to assess the prognostic value of PGCA-derived mutations. Using a Cox proportional hazards model, we identified five genes—ADAMTS7, CDH1, DRD5, MGAT5, and TP53—whose mutations were significantly associated with biochemical recurrence (**Figure 5A**). These five genes were initially identified as somatically mutated in the PGCA component and subsequently validated for prognostic relevance using the prostate_dkfz_2018 dataset due to its larger sample size and clinical annotation. Functional annotation of these five genes revealed diverse roles in tumor progression. ADAMTS7 is involved in extracellular matrix remodeling and promotes metastasis (25). CDH1, encoding E-cadherin, regulates epithelial adhesion, and its loss facilitates epithelial–mesenchymal transition (EMT) (26, 27). DRD5, a dopamine receptor, is implicated in immune modulation and cell signaling (28). MGAT5 contributes to aberrant glycosylation promoting invasion and immune evasion (29). TP53 is a master regulator of DNA damage response and apoptosis, and its mutation disrupts genome stability and enables therapeutic resistance (30). The PFS analysis was performed to examine the correlation of candidate gene mutations and clinical recurrence, exhibiting significantly reduced recurrence-free rates in mutation carriers (**Figure 5B**). These findings were also validated in an alternative PCa cohort from public dataset prad_tcga, where mutation carriers exhibited significantly shorter recurrence-free survival (**Figure 5C**). Furthermore, we analyzed the OS rates of patients harboring mutations in the identified genes with the prad_mcspc_mskcc_2020 and prad_pik3r1_msk_2021 cohorts and found a significantly shorter OS compared with wild-type counterparts (**Figure 5D**). Together, these findings explicitly emphasized the prognostic value of identified mutation signature in PGCA.

Enormous evidence has demonstrated that prognostic factors generally participated in malignant progression. We therefore evaluated the potential associations of candidate gene mutations with pathology grades (Gleason score) and metastatic status. Stratified analysis by Gleason score revealed that mutation carriers were significantly enriched in high-grade tumors (Gleason score ≥ 8) across multiple cohorts, suggesting a positive correlation of the identified gene mutations and pathology grades (**Figures 5E, F**). Additionally, the mutation carriers exhibited notably higher frequencies of metastasis, and the gene mutations were mostly enriched in metastatic samples, suggesting a potential role for these mutations in driving metastatic progression (**Figures 5G, H**). These clinical correlation analyses with multiple cohorts revealed that mutations in ADAMTS7, CDH1, DRD5, MGAT5, and TP53 significantly correlated with and potentially responsible for the malignant progression and therapeutic resistance.

### Transcriptional validation of candidate gene mutations in malignant progression

**Figure 6:**
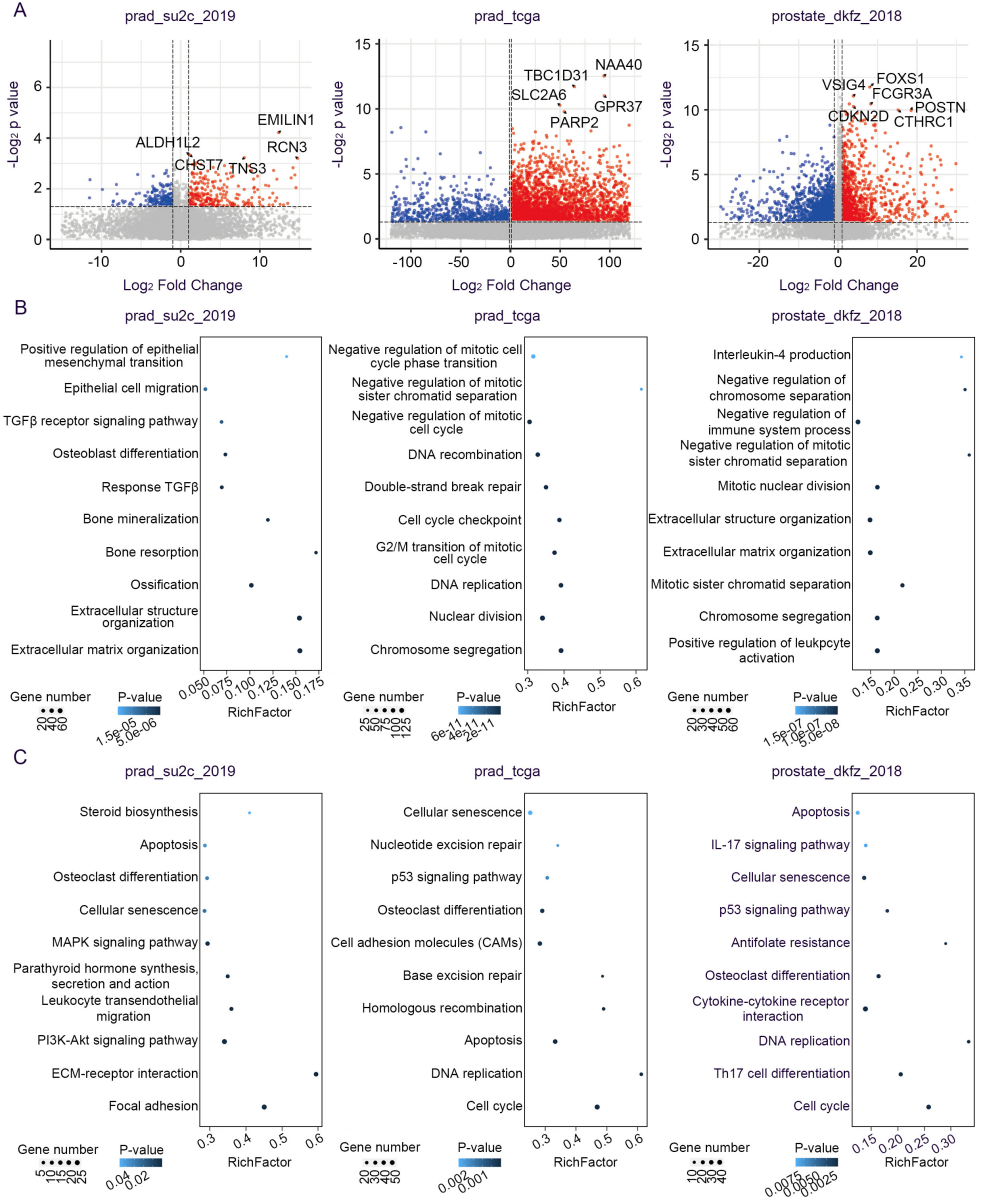
Transcriptomic alterations associated with signature mutations. **(A)** Volcano plot showing the DEGs between mutation-positive and mutation-negative tumors. **(B)** Biological processes and **(C)** signaling pathways of upregulated genes in PCa patients with signature mutations.

To delineate the molecular landscape of PCa driven by PGCA prognostic mutations, we examined the differentially expressed genes (DEGs) between mutation carriers and non-carriers from several cohorts, including prad_su2c_2019, prad_tcga, and prostate_dkfz_2018 (**Figure 6A**). These datasets were specifically selected because they represent extensive, conventional PCa cohorts with rich genomic and clinical annotations, enabling robust comparative validation of PGCA-derived mutations in a broader oncological context. Although PGCA is a rare and aggressive variant, evaluating its candidate mutations within these larger, conventional PCa cohorts allowed us to investigate the wider relevance and prognostic potential of these alterations beyond the rare subtype. Patients with candidate gene mutations highly expressed genes related to cell-cycle arrest and epithelial–mesenchymal transition (EMT), such as negative regulation of mitotic cell-cycle phase transition, negative regulation of mitotic sister chromatid separation, positive regulation of EMT, and extracellular matrix organization (**Figure 6B**). These processes were consistent with the typical PGCA features including the presence of multinucleated cells and enhanced metastatic potentials. Furthermore, we found that the mutation carriers showed high expression levels of genes enriched in IL-4 production and negative regulation of the immune system process, suggesting an immunosuppressive potential for tumor growth (**Figure 6B**). On the other hand, KEGG signaling pathway enrichment analysis showed that mutation carriers exhibited aberrant activation of p53 and PI3K-Akt signaling pathway, both critical for survival and therapeutic resistance, and dysregulation of biological processes including DNA replication, cell cycle, and homologous recombination, collectively contributing to genomic instability (**Figure 6C**). Concurrently, integration of multicohort transcriptomic data revealed that PGCA candidate mutations drive transcriptional reprogramming mainly related to cell-cycle dysregulation, metastasis, and anti-apoptosis.

## Discussion

Emerging evidence indicated pleomorphic giant cells with polyploidy in tumors associated with drug resistance and adverse survival (31). In this study, a prostatic PGCA patient administered with chemotherapy and endocrinotherapy alongside TURP only lived for 6 months post-diagnosis. Histopathology examinations showed a distinct phenotype characterized by cellular pleomorphism, multinucleated giant cells, and necrosis. Cellular pleomorphism of PGCA indicated significant malignancy of tumor cells, given that conventional adenocarcinoma, even with a high Gleason score, typically consists of cells with relatively uniform nuclei (32). Most multinucleated giant cells, induced by cell-cycle arrest, cell–cell fusion, or therapeutic stress, were identified around necrotic areas probably caused by the hypoxic microenvironment (33, 34). Furthermore, abundant studies have shown that the pleomorphic giant cells exhibited stem cell-like features with a similar proliferation cycle to embryonic development and dominated tumor metastasis (35–38). The unique immunohistochemical profiles examined from our study showed the absence of AR, AMACR, and KLK3 expression, coupled with high VIM expression in pleomorphic giant cells. Loss of AR and the downstream gene expression indicated insensitivity to androgen, suggesting resistance to endocrinotherapy, which was reported before in individual cases (8). A large number of studies showed that an elevated expression of VIM suggested a loss of epithelial differentiation and acquisition of mesenchymal traits, potentially linked to EMT and metastatic behaviors (39, 40). These findings demonstrated the potentially critical roles of pleomorphic giant cells in multiple drug resistance, attributed to the embryonic morphology, loss of AR, and overexpression of VIM.

The mutational landscape of PGCA components, examined by WES with samples obtained from LMD, showed significant enrichment in cell-cycle dysregulation and growth factor response with potential correlation with apoptosis. Among the mutated genes, TP53 and CDC25B have been verified capable of preventing G2/M-phase cell-cycle arrest, suggesting that the multinucleated giant cells were induced by cell-cycle arrest in PGCA (41–43). Early studies indicated that TGFβ and TNF were critical factors responsible for inflammatory microenvironment construction in malignant tumors and were also involved in a variety of cellular processes, including proliferation, motility, and apoptosis (44, 45). Mutations in genes associated with these signaling pathways could potentially cause apoptotic effects on tumor cells and promoted survival from aggressive treatments and the immune system in PGCA. In comparison, the coexisting adenocarcinoma also possessed TP53 mutation, among other gene mutations associated with TP53-mediated apoptotic dysfunction and metabolic aberrations. These findings demonstrated that both PGCA and coexisting adenocarcinoma possessed TP53 mutation and presented tolerance to chemotherapy and endocrinotherapy. Given that the pleomorphic giant cells have been reported capable of generating daughter cells with even more aggressive phenotypes through asymmetric division (46, 47), we analyzed the development trajectory of these two malignant components to examine if they had a directive evolutionary relationship. The clonal evolution analysis showed that they shared the same clonal origin yet diverged during malignant progression, acquiring distinct genomic signatures. Such lineage bifurcation explained the clinical aggressiveness of prostatic PGCA and its resistance to conventional therapies, also reminding us the aggressive malignancy of coexisting adenocarcinoma.

Our clinical correlation analysis with multiple public cohorts identified a total of five genes, namely, ADAMTS7, CDH1, DRD5, MGAT5, and TP53, whose mutations were significantly associated with malignant progression and adverse prognosis in PCa. TP53 mutations, well-documented in various malignancies associated with enhanced genomic instability and therapeutic resistance, were identified in both PGCA and adenocarcinoma (48, 49). The remaining gene mutations were all PGCA specific, among which ADAMTS7 mutations were found associated with oncogenesis and dismal prognosis in a variety of cancers (50–52). ADAMTS7 is a cartilage oligomeric matrix protein-cleaving enzyme involved in extracellular matrix remodeling, whose family members are also linked to tumor progression by affecting the interplay between the malignant cells and local microenvironment (53). Notably, tumor-suppressor CDH1 (E-cadherin) mutations were frequently inactivated in metastatic tumors with immunosuppressive microenvironment (54–58), likely contributing to immune evasion and migrative phenotype of pleomorphic giant cells in this case. DRD5 is a G protein-coupled receptor with differential expression and mutational profiles in various cancers. In 12 tumor types including bladder cancer, breast cancer, esophageal squamous cell carcinoma, and head and neck cancer, hypermethylation in the DRD5 promoter region leads to gene expression silencing, whereas in non-small cell lung cancer, DRD5 expression was elevated in response to docetaxel (59, 60). Activation of DRD5 by the selective agonist SKF83959 upregulates reactive oxygen species (ROS) levels in tumor cells, thereby activating the mitochondrial apoptosis pathway while inhibiting the mTOR pathway to induce autophagic cell death (28). However, there were also reports indicating that mutations or suppression of DRD5 could induce an immunocompromised microenvironment and promote cell survival from therapeutic drugs (28, 61, 62). MGAT5 encoding a glycosyltransferase was found overexpressed in a variety of malignancies, unlike our findings, whose loss function was noted for tumor suppression and inhibition of metastasis (63–66), implicating different roles for PGCA pathogenesis. In ovarian cancer, upregulation of MGAT5 could also exacerbate resistance to anti-PD-L1 immunotherapy by catalyzing branched N-glycans, which enhanced its binding with PD-1 on CD8^+^ T cells (67). Furthermore, transcriptional profiling confirmed that mutations in these genes drive transcriptional reprogramming linked to cell-cycle dysregulation, antiapoptosis, immunosuppression, and metastasis, reinforcing their functional relevance to the malignant maintenance of PGCA phenotypes.

This study is based on a single well-characterized case, which may limit broad generalization. However, given the extreme rarity of prostatic PGCA, our comprehensive multiomics and pathological profiling offers valuable insights and establishes a foundation for future studies in larger cohorts.

## Conclusion

This study provides the first integrative characterization of prostatic pleomorphic giant cell adenocarcinoma (PGCA), revealing its distinct histological, immunohistochemical, and genomic features. We identified key mutations—particularly in ADAMTS7, CDH1, DRD5, MGAT5, and TP53—associated with malignant progression and poor prognosis. PGCA showed transcriptional reprogramming related to cell-cycle dysregulation, immune evasion, and therapeutic resistance. Our findings uncover novel molecular insights into PGCA pathogenesis and suggest potential biomarkers and therapeutic targets for this rare and aggressive prostate cancer subtype.

## Data Availability

The WES data of this study is available in China National Center for Bioinformation / National Genomics Data Center under the accession code HRA010637. The VCF files containing mutational information has been deposited in National Genomics Data Center (https://ngdc.cncb.ac.cn/) under the accession number of subPRO062622.
